# Loss of zinc-finger protein 212 leads to Purkinje cell death and locomotive abnormalities with phospholipase D3 downregulation

**DOI:** 10.1038/s41598-021-02218-x

**Published:** 2021-11-23

**Authors:** Rin Khang, Areum Jo, Hojin Kang, Hanna Kim, Eunsang Kwag, Ji-Yeong Lee, Okjae Koo, Jinsu Park, Hark Kyun Kim, Dong-Gyu Jo, Inwoo Hwang, Jee-Yin Ahn, Yunjong Lee, Jeong-Yun Choi, Yun-Song Lee, Joo-Ho Shin

**Affiliations:** 1 Department of Pharmacology, Samsung Biomedical Research Institute, Sungkyunkwan University School of Medicine, Suwon, 16419 South Korea; 2 Single Cell Network Research Center, Sungkyunkwan University School of Medicine, Suwon, 16419 South Korea; 3 Laboratory Animal Research Center, Samsung Biomedical Research Institute, Sungkyunkwan University School of Medicine, Suwon, 16419 South Korea; 4 School of Pharmacy, Sungkyunkwan University, Suwon, 16419 South Korea; 5 Biomedical Institute for Convergence, Sungkyunkwan University, Suwon, 16419 South Korea; 6 Department of Molecular Cell Biology, Samsung Biomedical Research Institute, Sungkyunkwan University School of Medicine, Suwon, 16419 South Korea; 7 Samsung Biomedical Research Institute, Samsung Medical Center, Seoul, 06351 South Korea; 8 Present Address: ToolGen, Seoul, 08501 South Korea

**Keywords:** Neurodegeneration, Movement disorders

## Abstract

Although Krüppel-associated box domain-containing zinc-finger proteins (K-ZNFs) may be associated with sophisticated gene regulation in higher organisms, the physiological functions of most K-ZNFs remain unknown. The Zfp212 protein was highly conserved in mammals and abundant in the brain; it was mainly expressed in the cerebellum (Cb). Zfp212 (mouse homolog of human ZNF212) knockout (Zfp212-KO) mice showed a reduction in survival rate compared to wild-type mice after 20 months of age. GABAergic Purkinje cell degeneration in the Cb and aberrant locomotion were observed in adult Zfp212-KO mice. To identify genes related to the ataxia-like phenotype of Zfp212-KO mice, 39 ataxia-associated genes in the Cb were monitored. Substantial alterations in the expression of ataxin 10, protein phosphatase 2 regulatory subunit beta, protein kinase C gamma, and phospholipase D3 (*Pld3*) were observed. Among them, *Pld3* alone was tightly regulated by Flag-tagged ZNF212 overexpression or Zfp212 knockdown in the HT22 cell line. The Cyclic Amplification and Selection of Targets assay identified the TATTTC sequence as a recognition motif of ZNF212, and these motifs occurred in both human and mouse PLD3 gene promoters. Adeno-associated virus-mediated introduction of human ZNF212 into the Cb of 3-week-old Zfp212-KO mice prevented Purkinje cell death and motor behavioral deficits. We confirmed the reduction of Zfp212 and Pld3 in the Cb of an alcohol-induced cerebellar degeneration mouse model, suggesting that the ZNF212–PLD3 relationship is important for Purkinje cell survival.

## Introduction

The Krüppel-associated box (KRAB) domain-containing zinc-finger protein (K-ZNF) group is the largest transcription regulator family and is specifically expressed in tetrapods^1^. ZNF protein contains a zinc-finger domain that can bind to DNA, RNA, proteins, and small molecules. ZNF proteins usually contain multiple zinc-finger domains and other functional domains, which make ZNF the most diverse protein family in higher organisms. ZNFs function as different regulators in the cell, and K-ZNFs mainly function as transcription factors^2^.

The human genome encodes approximately 350 K-ZNFs^3^. The functions of most K-ZNFs have not yet been determined, and many studies have focused on understanding their genomic information throughout evolution. Notably, emerging research on K-ZNFs has revealed that several K-ZNFs play an important role in neurophysiology and neurodegenerative diseases. For example, we previously identified ZNF746/PARIS as a parkin-interacting substrate that suppresses mitochondrial biogenesis in dopaminergic neurons (DA), thus leading to DA degeneration in Parkinson’s disease (PD) pathogenesis^4,5^. ZNF238/Rp58 is involved in neocortex development and is crucial for cerebellar growth and organization and early development of GABAergic and glutamatergic cerebellar neurons^6–8^. Genome-wide association studies have shown that many ZNF genes are also associated with mental illnesses, such as schizophrenia, bipolar disease, and intellectual disability^9^.

Hence, in this study, we characterized a novel ZNF212 encoded on human chromosome 7, a nearby cluster of several other KZNFs, including ZNF282, ZNF783, ZNF777, ZNF398, ZNF596, and ZNF746. Notably, Zfp212 (a mouse homolog of human ZNF212) is highly expressed in the cerebellum (Cb). To explore the functions of ZNF212 in vivo, we generated mice with the Zfp212 gene knockout (KO) allele. Deletion of Zfp212 resulted in cerebellar Purkinje cell death, followed by ataxia-like movement disorders.

Zfp212-KO mice have an ataxia-like movement disorder. We explored the mRNA level of 39 ataxia-related genes in wild-type (WT) and Zfp212-KO mice Cb by reverse transcription quantitative real-time polymerase chain reaction (RT-qPCR) analysis. RT-qPCR revealed that the mRNA transcription levels of four ataxia-related genes (ataxin 10 (*Atxn10*), protein phosphatase 2 regulatory subunit B beta (*Ppp2r2b*), protein kinase C gamma (*Prkcg*), and phospholipase D3 (*Pld3*)) were significantly changed in the Zfp212-KO Cb compared to those in the WT. PLD3 was finally identified as a target gene of ZNF212 by Flag-tagged ZNF212 overexpression or Zfp212 knockdown in the hippocampal neuron cell line HT22, which was followed by a promoter assay. As a result, we concluded that the reduction of *Pld3* in the absence of Zfp212 leads to Purkinje cell death and movement disorder.

The mammalian PLD family comprises the following three genes: *PLD1*, *PLD2*, and *PLD3*. PLD families contain the HxKxxxxD/E (where x is any amino acid residue) motif. PLD3 lacks Phox and pleckstrin homology domains, which are involved in the membrane targeting of PLD, resulting in the loss of PLD activity^10,11^. PLD3 is highly expressed in the brain, especially in neurons, but at a lower level in non-neuronal tissues. Whole exome sequencing (WES) of 14 late-onset Alzheimer’s disease (LOAD) families and confirmation analysis of several large LOAD cases revealed Val232Met variation in *PLD3*, implying that rare coding variants of *PLD3* might increase the risk of LOAD^12^. In addition, PLD3 expression was downregulated in the AD brain and exhibited a negative correlation with amyloid precursor protein (APP) and amyloid-β (Aβ) levels. Accordingly, PLD3 knockdown increased APP and Aβ levels, whereas PLD3 overexpression significantly decreased the levels of APP and Aβ, demonstrating that the homeostasis of PLD3 may be important for preventing neurodegeneration in AD.

Of note, WES of 20 spinocerebellar ataxia (SCA) families, who remained undiagnosed after regular DNA diagnostics, also identified *PLD3* as a novel ataxia gene^13^. The Leu308Pro mutation of *PLD3* was found in an independent SCA cohort, indicating that abnormal PLD3 can be a determinant of SCA^13^. The enzymatic function of PLD3 is currently unclear^14^, and how the loss or mutation of PLD3 in neuronal cells leads to SCA is also unknown. However, a recent study revealed that PLD3 is processed into a soluble form and stably resides within endosomes and lysosomes^15^. The stabilized form of PLD3 in the lysosome may contribute to lysosomal function to break down the plaques in neurons, providing the possibility of revealing the mechanism for maintaining neuronal health in SCA. Taken together, the expression of ZNF212 might be crucial for Purkinje cell survival and locomotion in mice.

## Results

### ZNF212 is expressed in the Purkinje cells

**Figure 1:**
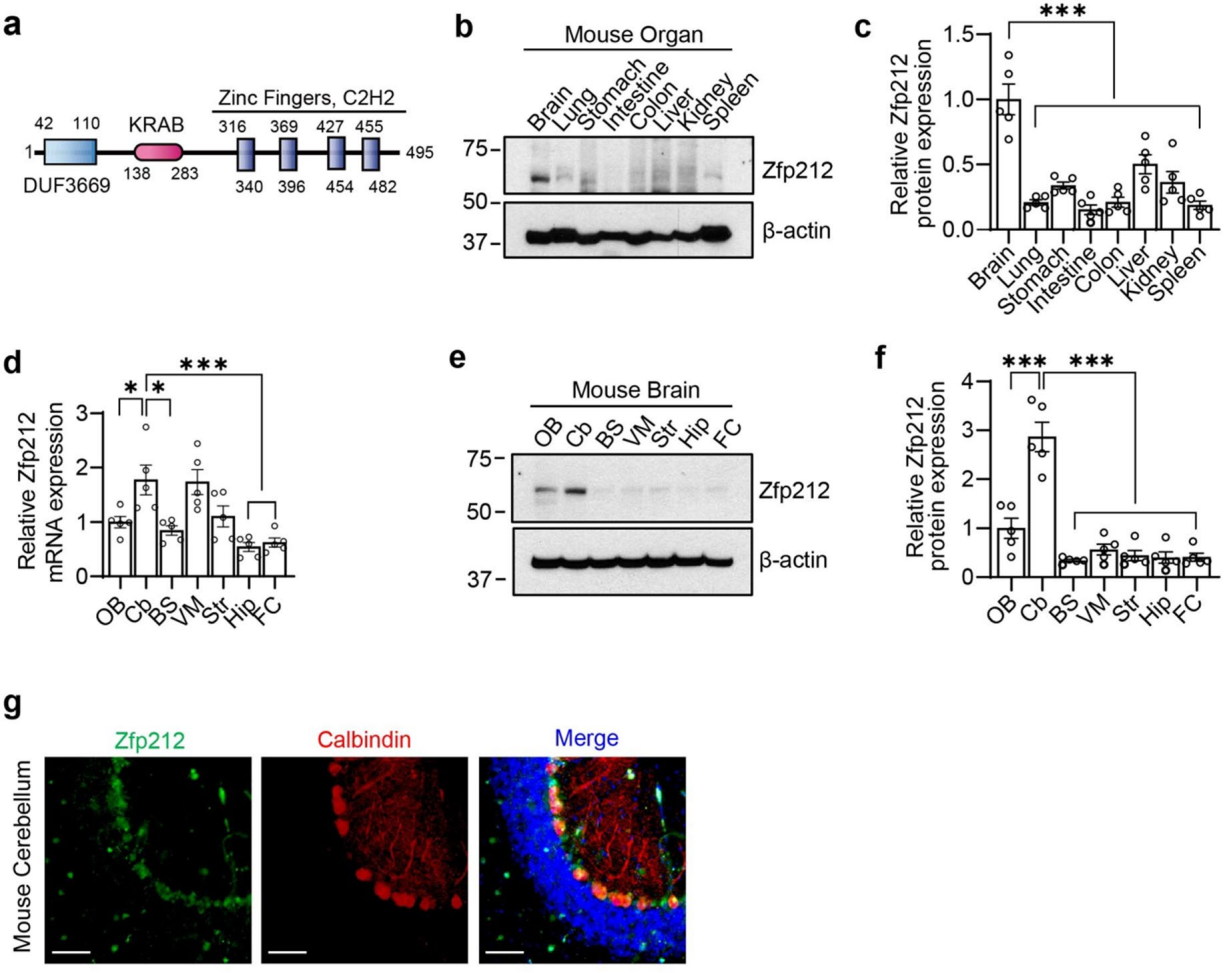
Zfp212 is expressed in the cerebellum. (**a**) Functional domains of human ZNF212. ZNF212 is composed of DUF3669, KRAB, and C2H2 zinc-finger domains. (**b**, **c**) Expression profile of Zfp212 in mouse organs. Zfp212 is highly expressed in the brain. Quantification of immunoblots (**c**, *n* = 5). (**d**) RT-qPCR analysis of Zfp212 in the sub-regions of the brain (OB; olfactory bulb, Cb; cerebellum, BS; brain stem, VM; ventral midbrain, Str; striatum, Hip; hippocampus, and FC; frontal cortex). Relative mRNA levels were normalized to the reference gene Rpl32 (*n* = 5). (**e**, **f**) Immunoblot of Zfp212 in sub-regions of the mouse brain. Quantification of immunoblots (**f**, *n* = 5). (**g**) Immunofluorescence of ZNF212 and calbindin (Purkinje cell marker) in the lobule V of mouse cerebellum. Scale bars = 50 μm.

ZNF212 is a KRAB domain-containing ZNF. ZNF212 is composed of three functional domains: DUF3669, KRAB, and C2H2 zinc-finger domains (Fig. 1a). In general, the C2H2 zinc-finger domain binds to DNA, and the KRAB domain represses the transcriptional target. However, the function of the DUF3669 domain is currently unknown. Multiple sequence alignment of mammalian ZNF212/Zfp212 (mouse homolog of human ZNF212) shows that the amino acid sequences of mammalian ZNF212 are highly conserved among species (Fig. S1). We conducted immunofluorescence staining of ZNF212 in HEK293 cells and found that ZNF212 was predominantly located in the nucleus (Fig. S2a). Next, we validated the specificity of ZNF212 antibody in HEK293 cells transfected with siRNA-ZNF212 (Fig. S2b) and investigated the protein levels of Zfp212 in various mouse organs (the brain, lungs, stomach, intestines, colon, liver, kidneys, and spleen); Zfp212 was robustly expressed in the brain (Fig. 1b, c).

Furthermore, we determined the expression pattern of Zfp212 in the sub-regions of the mouse brain (olfactory bulb (OB), Cb, brain stem (BS), ventral midbrain (VM), striatum (Str), hippocampus (Hip), and frontal cortex (FC); Fig. 1d–f). Zfp212 mRNA was transcribed in the various brain region and its levels were slightly higher in the Cb and VM (Fig. 1d). Immunoblot analysis showed robust expression of Zfp212 in the Cb and OB (Fig. 1e, f). Immunostaining analysis showed that Zfp212 co-localized with calbindin, a marker of Purkinje cells in the cerebellar lobule V of 8-week-old male mice (Fig. 1g), suggesting that Zfp212 might play a key role in cerebellar function. Next, we monitored the cerebellar level of Zfp212 in the different developmental stages of the mouse cerebellum, revealing that cerebellar Zfp212 was minimally expressed at embryonic day 18 (E18) and postnatal day 0 whereas robust expression was observed at 3 weeks of age and its level was decreased after 5 weeks of age (Fig. S2c). These results suggest that cerebellar Zfp212 might be important for Purkinje neuronal maintenance at postnatal 3 weeks.

### Adult Zfp212-KO mice show Purkinje cell death

To understand the importance of ZNF212 in cerebellar physiology, we generated *Zfp212*-KO mice using CRISPR/Cas9 (Fig. S3). Recombinant Cas9 protein and two gRNAs were microinjected into fertilized mouse zygotes and inserted into surrogate mothers. The genotypes of the newborn mice were identified by PCR and sequencing (Fig. S3a–d). Three Zfp212-KO mouse lines (Δ1, Δ176, + 1) were produced, and the Δ176 line was mainly utilized in this study because of the convenience of maintenance (Fig. S3e, f). The survival rate of Zfp212-WT, -hetero (Het), and -KO mice showed that the loss of Zfp212 did not cause significant death at a young age (Fig. S3g). However, 40% of the Zfp212-KO mice died at approximately 22 months of age compared to the WT and Het mice (Fig. S3g).

**Figure 2:**
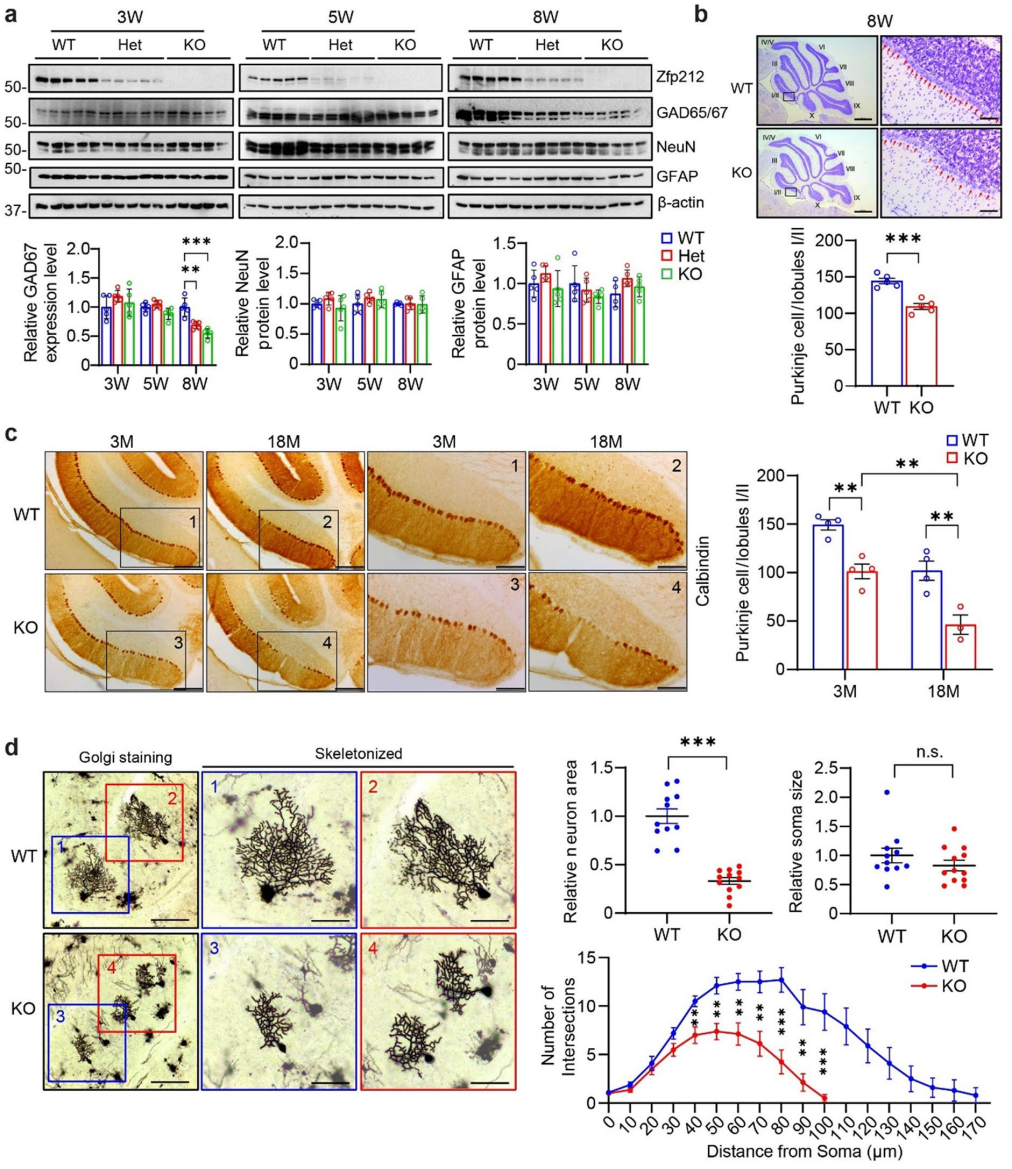
Adult Zfp212-KO mice show cerebellar Purkinje cell death. (**a**) Immunoblot analysis of Zfp212, GAD65/67, NeuN, and GFAP in Zfp212-WT, -Het, and -KO cerebella at three different time points (3, 5, and 8 weeks of age). Lower panels are the quantification of the immunoblot (3 weeks: *n* = 5 per group, 5 weeks: *n* = 5 per group, 8 weeks: *n* = 5 per group). (**b**) Representative image of Nissl staining in the cerebellum of Zfp212-WT and -KO mice at 8 weeks of age. The number of Purkinje cells in lobules I/II was counted (at the bottom of panel **b**, *n* = 5). Scale bars = 800 μm at the original image and 80 μm at the enlarged image, respectively. (**c**) Representative image of DAB staining for calbindin in the cerebellum of Zfp212-WT and -KO mice. Quantification of Purkinje cells/lobules I/II is shown on the right (*n* = 3). Scale bars = 200 μm at the original image and 100 μm at the enlarged image, respectively. (**d**) Golgi staining of Purkinje cells and their skeletonized images in Zfp212-WT and -KO cerebella at 3 months of age (WT: *n* = 11, KO: *n* = 11). Scale bars = 100 μm at the original image and 50 μm at the enlarged image, respectively. n.s., not significant.

Since Zfp212 is abundant in calbindin-positive Purkinje cells of Cb, we monitored the levels of GAD65/67 (GABAergic neuronal marker), NeuN (neuronal marker), and GFAP (glial marker) in the Cb of Zfp212-WT and KO mice at 3, 5, and 8 weeks of age (Fig. 2a). Notably, the levels of NeuN and GFAP were comparable in all groups. In contrast, GAD65/67 was dramatically decreased in the Cb of 8-week-old Zfp212-Het and KO mice but not in that of younger Zfp212-Het and KO mice, suggesting that ZNF212 is required for the integrity of Purkinje cells at the adult stage (Fig. 2a). In addition, Nissl staining of Cb of 8-week-old Zfp212-WT and KO mice showed a significant loss of Purkinje neurons in the lobules I/II of 8-week-old Zfp212-KO cerebellum (Fig. 2b). To assess the degeneration of Purkinje cells in the absence of Zfp212, Nissl staining of Cb sections of 3-month-old Zfp212-WT and KO mice were compared, which indicated that the number of Purkinje cells was reduced in the motor-associated lobules I/II, III, IV/V, and VIII as well as nonmotor lobules VI and IX/X of Zfp212-KO mice compared to that of WT mice (Fig. S4a). Immunohistochemical staining confirmed that the number of calbindin-positive Purkinje cells declined by 30% and 50% in the Cb of 3- and 18-month-old Zfp212-KO mice, respectively (Fig. 2c). Immunofluorescence with calbindin and NeuN antibodies also showed reduced numbers and loose and shorter dendrites of Purkinje cells in the Cb of 18-month-old Zfp212-KO compared to WT, whereas NeuN-positive granular cells were comparable between Zfp212-WT and KO mice (Fig. S4b).

To determine the morphological degeneration of Purkinje cells in Zfp212-KO mice, we applied Golgi staining and skeletonized images for quantification (Fig. 2d). The neuronal area of Purkinje cells in the Cb of Zfp212-WT was significantly larger than that of Zfp212-KO, whereas the soma size was comparable between groups. Dendrite Sholl analysis showed that dendrites of Zfp212-KO Purkinje cells were significantly shorter than those of WT. Taken together, Zfp212 loss leads to a reduced number and morphological abnormalities of Purkinje cells in the Cb (Fig. 2d).

### Abnormal locomotion in Zfp212-KO mice

**Figure 3:**
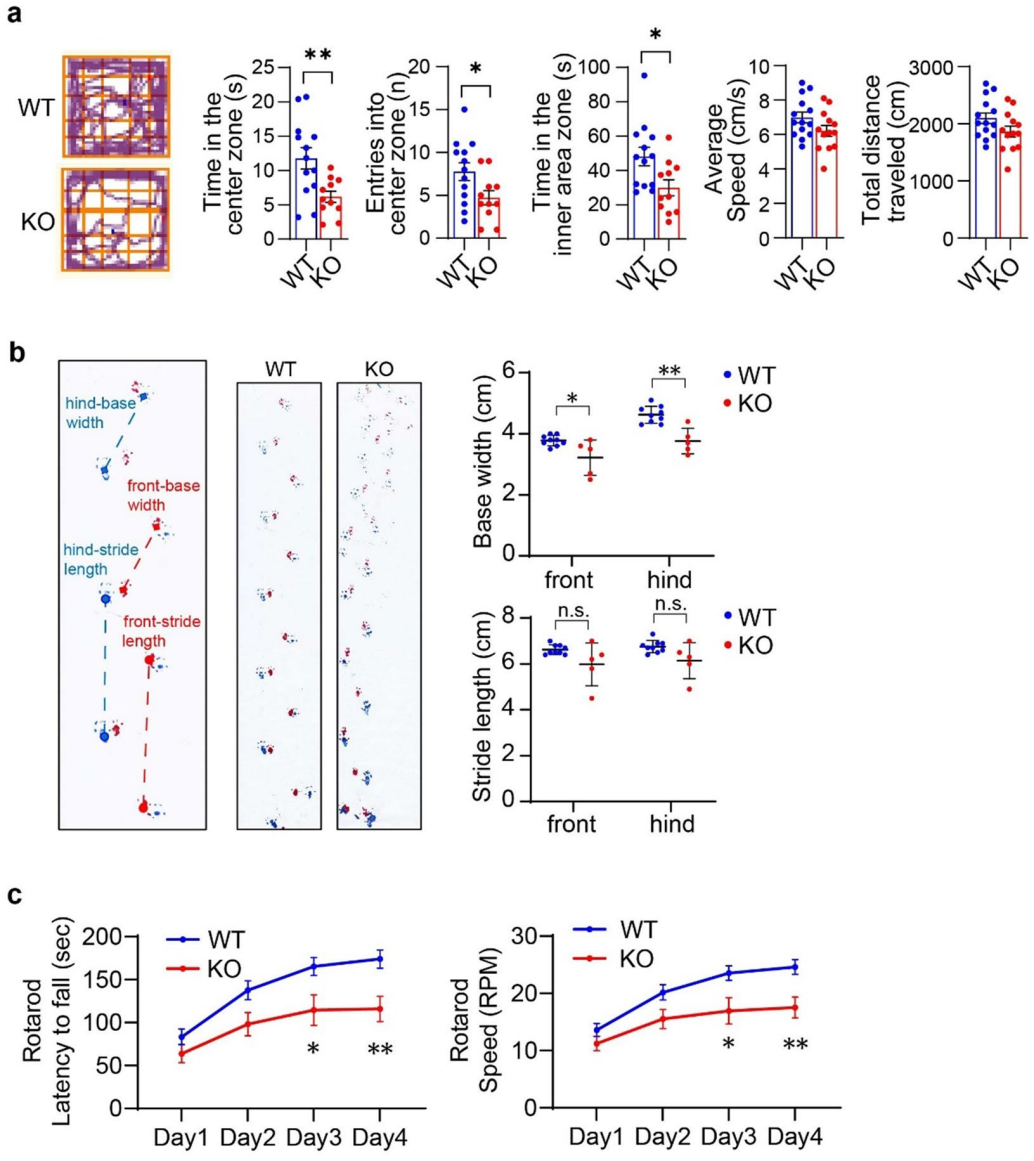
Adult Zfp212-KO mice show aberrant locomotion. (**a**) Open-field test of 3-month-old Zfp212-WT and -KO mice (WT; *n* = 13, KO; *n* = 9). Movement of mice was tracked and analyzed with EthoVision XT software (https://www.noldus.com/ethovision-xt). (**b**) Gait analysis of 3-month-old Zfp212-WT and -KO mice (WT, *n* = 9; KO, *n* = 5). n.s., not significant. (**c**) Rotarod test of 3-month-old Zfp212-WT and -KO mice (WT; *n* = 13, KO; *n* = 9).

To investigate whether Purkinje cell death implicates locomotion ability at the adult stage of Zfp212-KO mice, we conducted behavioral tests with 9-week-old Zfp212-WT and KO mice. The open-field test showed that the average speed and total distance traveled were comparable between Zfp212-WT and KO mice, whereas Zfp212-WT mice entered the center zone more often and spent more time there than KO mice, indicating that Zfp212-KO mice exhibit anxiety-like behavior (Fig. 3a).

To validate whether Zfp212-KO mice show a cerebellar-related behavioral abnormality, gait analysis was performed using 9-week-old Zfp212-WT and KO mice. Both front and hind base widths significantly decreased in Zfp212-KO mice compared to WT mice, and there was a trend of decreasing stride lengths in Zfp212-KO mice. Footprints demonstrated gait dysfunction in Zfp212-KO mice (Fig. 3b).

Finally, we applied the Rotarod test to assess the locomotor and balance deficits of Zfp212-KO mice, which revealed that Zfp212-KO mice performed less well than WT mice, spending less time on the rod before falling. A similar result was obtained for the speed at which Zfp212-KO mice fell from the rod, which was slower than that for WT mice during the training phase (Fig. 3c). Taken together, Zfp212-KO mice showed anxiety-like behavior and locomotor dysfunction.

### ZNF212 regulates ataxia-related genes in the cerebellum

**Figure 4:**
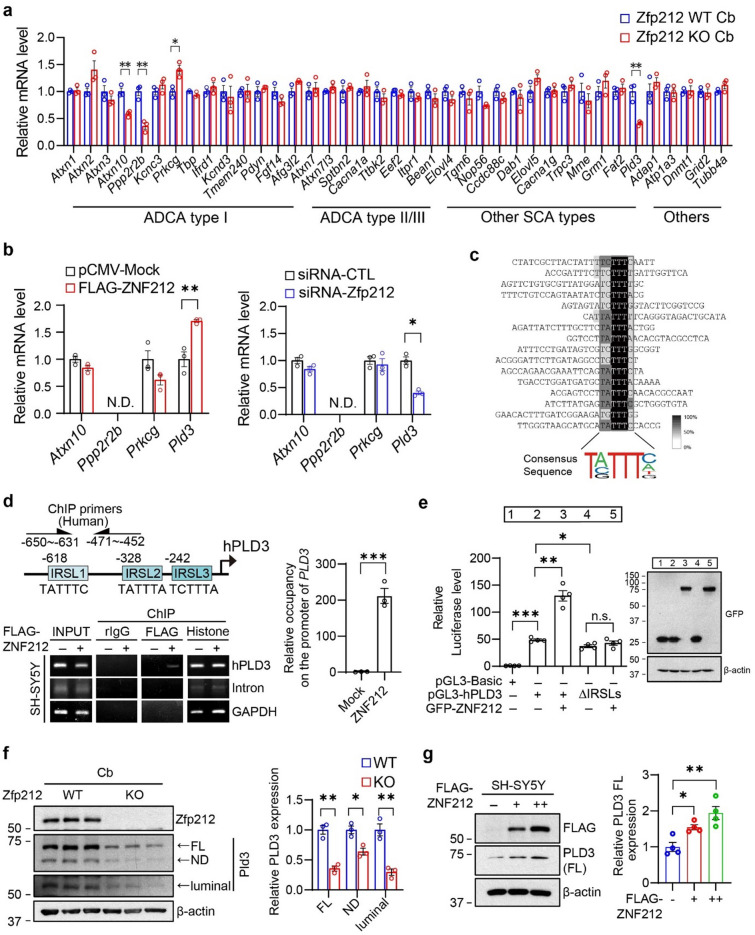
ZNF212 regulates PLD3 by binding to the insulin responsive sequence-like motif. (**a**) RT-qPCR analysis of 39 cerebellar ataxia-related genes in the cerebellum of 3-month-old Zfp212-WT and -KO mice. Relative mRNA levels were normalized to reference genes, Rpl32 (*n* = 3). (**b**) mRNA levels of *Atxn10*, *Pp2r2b*, *Prkcg*, and *Pld3* in HT22 cells transfected with Flag-tagged ZNF212 or siRNA-Zfp212 (*n* = 3). N.D., not detected. (**c**) CAST analysis with GST-tagged ZNF212, identifying IRSL (insulin responsive sequence-like) motif as a DNA binding site of ZNF212. Sixteen ZNF212-bound DNA probes were sequenced and aligned with the Multiple Alignment Construction and Analysis Workbench (MACAW 2.05, https://www.en.bio-soft.net/format/MACAW.html) software. (**d**) Schematic image of human *PLD3* promoter containing IRSLs. ChIP assay with SH-SY5Y cells overexpressing Flag-tagged ZNF212 Rabbit IgG (rIgG) and histone antibodies used as negative and positive controls, respectively. Three replicated experiments were quantified with quantitative real-time PCR. ChIP elute was normalized with input. (**e**) Luciferase promoter assay with SH-SY5Y cells transfected with the human PLD3 promoter and GFP-tagged ZNF212. Transfection efficacy was confirmed by immunoblot analysis. n.s., not significant. (**f**) Immunoblot of Zfp212 and Pld3 in the cerebellum of 8-week-old Zfp212-WT and KO mice. Three forms of Pld3 were annotated using full-length (FL), N-terminal deletion (ND), and luminal Pld3. Pld3 expression was quantified (right panel, *n* = 3). (**g**) ZNF212 activated PLD3 in a dose-dependent manner. Quantification of PLD3 proteins in the right panel (*n* = 4).

To identify the target genes of ZNF212 associated with the behavioral phenotype of Zfp212-KO mice, we selected 39 ataxia-related genes^16,17^ and monitored their mRNA levels in the Cb of 8-week-old Zfp212-WT and KO mice (Fig. 4a). Significant alterations in the mRNA levels of *Atxn10*, *Ppp2r2b*, *Prkcg*, and *Pld3* were found in the Cb of Zfp212-KO mice compared to those in the Cb of WT mice (Fig. 4a). These four genes were subjected to further evaluation using the neuronal cell line HT22 transfected with Flag-tagged ZNF212 or siRNA-Zfp212, revealing that *Pld3* alone was regulated by ZNF212/Zfp212 overexpression or knockdown (Fig. 4b). The regulation of PLD3 protein by Zfp212 was confirmed in HT22 cells transfected with Flag-tagged ZNF212 or siRNA-Zfp212 (Fig. S5a, b). Inconsistencies in *Atxn10*, *Ppp2r2b*, and *Prkcg* mRNA profiles between the mouse Cb of Zfp212-KO and HT22 cells transfected with siRNA-Zfp212 might be attributed to the disturbance of non-neuronal cells in the Cb.

Moreover, the CAST assay identified the TATTTC sequence, similar to the insulin responsive sequence (IRS), as a recognition motif of ZNF212 (Fig. 4c), and these IRS-like sequences (IRSLs) were found in both human and mouse *PLD3* promoters (Figs. 4d and S6a). The ChIP assay confirmed that ZNF212 occupies the IRSL region of the *PLD3* promoter (Figs. 4d and S6b), and the luciferase reporter assay demonstrated that the overexpression of ZNF212 activated the *PLD3* promoter but not the IRSL-deficient *PLD3* promoter, suggesting that ZNF212 transcriptionally activates *PLD3* via IRSLs (Fig. 4e).

To evaluate the relationship between ZNF212 and PLD3 in vivo, we monitored the protein level of Pld3 in the Cb of Zfp212-WT and KO mice, showing that full-length and N-terminal deleted Pld3 (FL-Pld3 and ND-Pld2) were significantly downregulated by 60% and luminal Pld3 by 40% in the Cb of Zfp212-KO mice compared to WT mice (Fig. 4f). In addition, FL-PLD3 increased in a dose-dependent manner in SH-SY5Y cells transfected with Flag-tagged ZNF212 (Fig. 4g), and ND-PLD3 and luminal PLD3 were not detectable. Taken together, ZNF212 binds to the IRSLs of the *Pld3* promoter and transcriptionally activates *Pld3* in the mouse Cb.

### Delivery of human ZNF212 into the cerebellum of Zfp212-KO mice prevents Purkinje cell death and ataxia-like behavior

**Figure 5:**
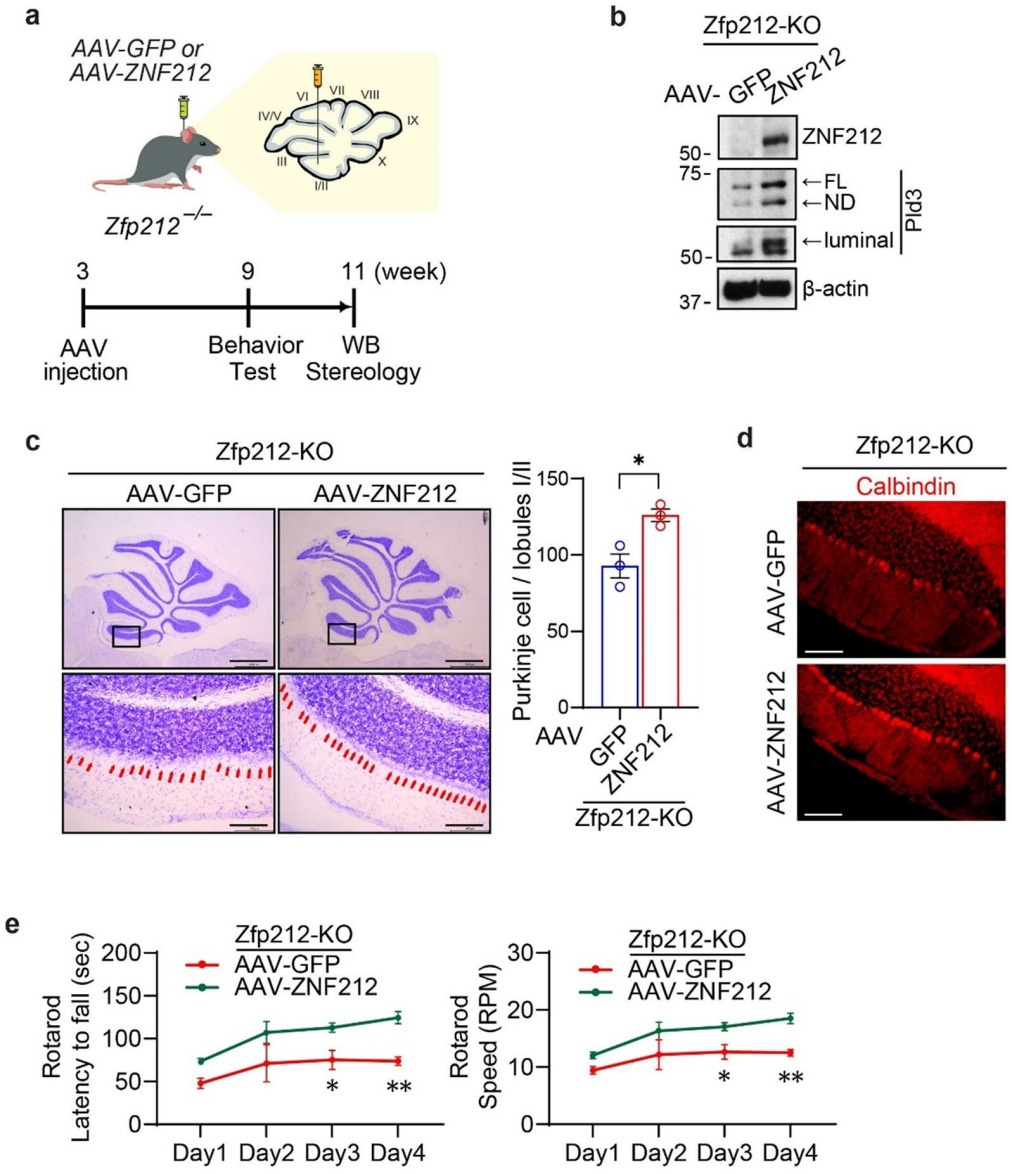
Delivery of human ZNF212 rescues Purkinje cell death and aberrant locomotion in Zfp212-KO mice. (**a**) Stereotaxic administration of AAV-GFP and AAV-ZNF212 into the cerebellum (lobule I/II) of 3-week-old Zfp212-KO mice. Behavioral tests and further analyses were performed at the indicated time points. (**b**) Overexpression of human ZNF212 in the cerebellum of Zfp212-KO mice was confirmed by immunoblot analysis. Expression was normalized to β-actin (bottom panel, *n* = 3). (**c**) Representative image of Nissl staining in the cerebellum of AAV-GFP and AAV-ZNF212 injected in Zfp212-KO mice 8 weeks post-injection. Scale bars = 1000 μm at the original image and 100 μm at the enlarged image, respectively. (**d**) Calbindin staining of the cerebellum of Zfp212-KO mice injected with AAV-GFP or AAV-ZNF212 at 8 weeks post-injection. Scale bars = 100 μm. (**e**) Rotarod test of Zfp212-KO mice injected with AAV-GFP or AAV-ZNF212 (AAV-GFP; *n* = 4, AAV-ZNF212; *n* = 5).

To validate whether the Purkinje cell death and behavioral abnormalities observed in Zfp212-KO mice can be restored by the introduction of exogenous human ZNF212, we stereotaxically injected adeno-associated virus (AAV)-ZNF212 into the lobules I/II of the Zfp212-KO Cb mice at 3 weeks of age (Fig. 5a). Accompanying the introduction of ZNF212 was the upregulation of Pld3 at 8 weeks post-injection (Fig. 5b). Nissl staining of Cb sections of Zfp212-KO mice injected with AAV-GFP or AAV-ZNF212 were compared, indicating that the number of Purkinje cells was restored in the Cb of Zfp212-KO mice injected with AAV-ZNF212 compared to that of Zfp212-KO mice injected with AAV-GFP in lobules I/II (Fig. 5c), which is responsible for locomotive ability. Immunostaining images showed that the reduced number of calbindin-positive Purkinje cells in lobules I/II of Zfp212-KO was restored by AAV-ZNF212 injection (Fig. 5d). Furthermore, the delivery of ZNF212 into lobules I/II of Zfp212-KO Cb mice partially improved their locomotion capabilities (Fig. 5e).

### Chronic alcohol intake leads to suppressed ZNF212 expression in the cerebellum

**Figure 6:**
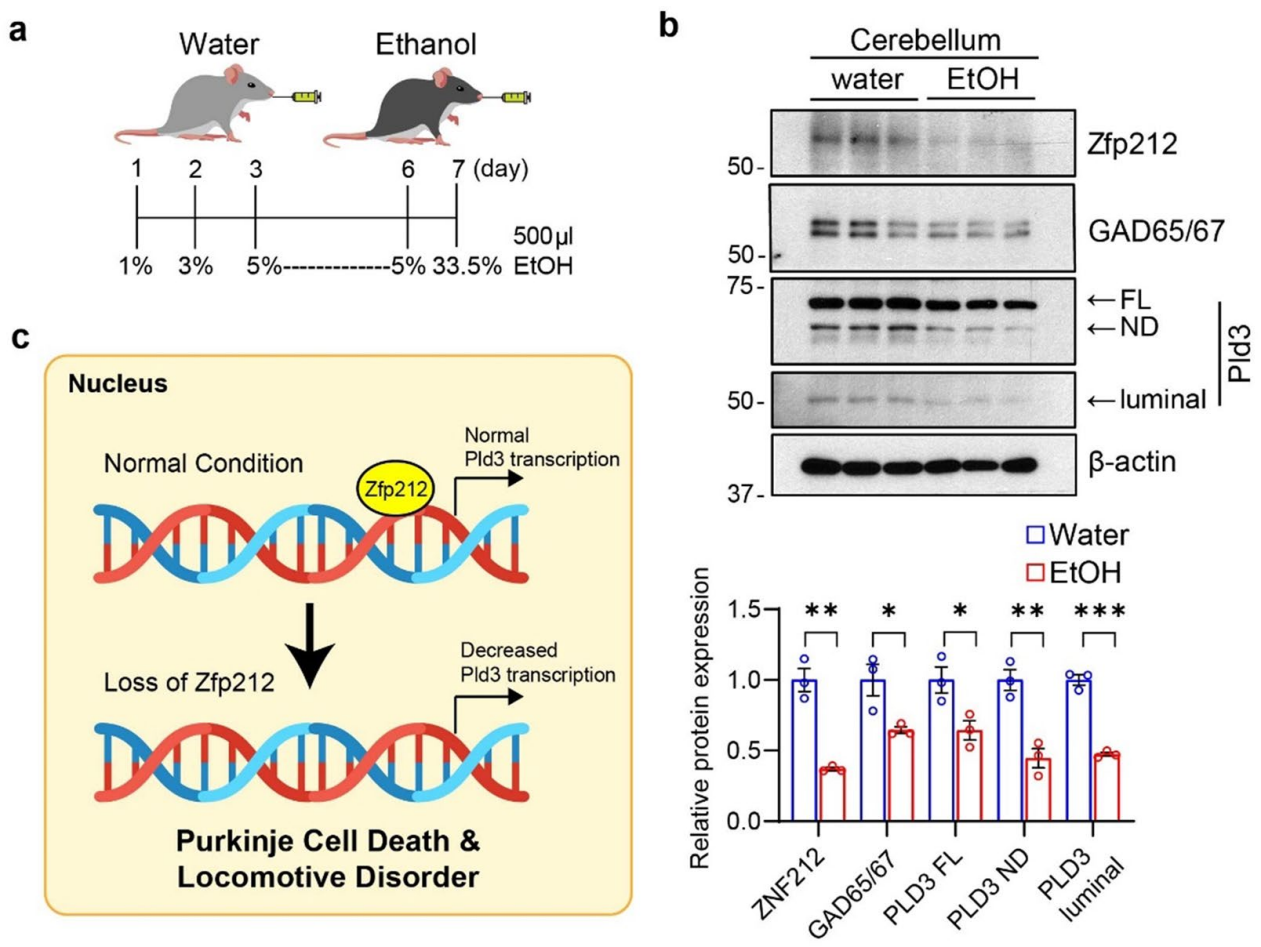
ZNF212 and PLD3 are downregulated in the cerebellum of alcohol-intake mice. (**a**) Timeline of ethanol administration in 8-week-old mice. The time and dose are indicated. (**b**) Protein levels of Zfp212, GAD65/67, and Pld3 in the cerebellum of alcohol-administered mice. Quantification of each protein was performed by β-actin normalization (bottom panel, *n* = 3). (**c**) Schematic image of Pld3 transcription regulation in Zfp212-KO Purkinje cells.

Since excessive alcohol exposure can cause cerebellar ataxia and locomotion dysregulation, such as impaired postural stability and balance as well as slower attenuated foot tapping, we assessed the levels of Zfp212, GAD65/67, and Pld3 in the Cb of mice administered 500 μL of alcohol (1–33.5%) per day for 1 week (Fig. 6a). In this alcohol-induced cerebellar atrophy model^18^, a significant reduction in Zfp212 was found in the Cb of alcohol-intake mice, accompanied by the downregulation of GAD65/67, FL-Pld3, ND-Pld3, and luminal Pld3 (Fig. 6b). These results suggest that the ZNF212-PLD3 pathway may be involved in alcohol-induced cerebellar dysfunction (Fig. 6c).

## Discussion

The human K-ZNF family comprises approximately 350 genes^1^. Most human K-ZNF ages are restricted to either primates or eutherians. K-ZNFs containing protein domain DUF3669 (domain of unknown function 3669) are considered ancient since they share origins with marsupials or sauropsids^19^. Therefore, the high conservation of ancient K-ZNFs may be due to their essential physiological roles. The genes of human K-ZNFs possessing DUF3669, including ZNF398, ZNF282, ZNF212, ZNF783, ZNF777, and ZNF746, reside in human chromosome 7^3^. K-ZNF clustering in the near region of the chromosome may imply that these K-ZNFs are involved in a similar functional category. Indeed, ZNF proteins play a functional role in maintaining brain physiology and are associated with neuronal disorders^4–9^. In the process of elucidating the functional role of K-ZNFs in the brain, we found that Zfp212 is highly expressed in the Cb and endogenously expressed in Purkinje cells. Zfp212-KO mice showed a significant decline in the level of GAD65/67, which is a GABAergic neuronal marker in the Cb in the adult stage, suggesting that Zfp212 is related to the maintenance of Purkinje cells in an age-dependent manner.

Since Zfp212-KO mice showed Purkinje cell death along with motor deficits, we monitored the levels of 39 ataxia genes in the Cb of Zfp212-KO mice. RT-qPCR data analysis revealed that *Atxn10*, *Ppp2r2b*, *Prkcg*, and *Pld3* were altered in the Cb of Zfp212-KO mice. Among them, PLD3 is tightly regulated by ZNF212 and is considered a reliable ZNF212 target protein. As described earlier, WES of 20 SCA families identified *PLD3* as a novel ataxia gene and found Leu308Pro (L308P) mutation in SCA families^13^, suggesting a possible mechanism by which abnormal PLD3 can be a determinant for SCA. Indeed, PLD3-WT was predominantly expressed in endolysosomes, whereas the PLD3-L308P mutant was mainly expressed in the endoplasmic reticulum (ER)^20^. Western blot analysis also showed that PLD3-WT has a cleavage luminal form, whereas L308P has only the membrane-bound full-length form. The results indicated that PLD3 is usually processed and trafficked from the ER into the endolysosome, but the L308P remains bound to the ER, leading to functional failure of PLD3 as a lysosomal protein. However, whole-body Pld3-KO mice did not show ataxia-like behavior at 20 months of age^20^. There might be a compensation for other lysosomal proteins during the development of germ-line Pld3-KO mice, counteracting the ataxia-like phenotype^21^. In contrast, the decrease in *Pld3* due to the loss of Zfp212 might contribute to endolysosomal dysfunction and Purkinje cell death.

Next, administration of human ZNF212 into the cerebellar lobules I/II of Zfp212-KO upregulated the level of Pld3 and rescued Purkinje cell death in the lobules I/II of Zfp212-KO mice, contributing to motor behavioral restoration in Zfp212-KO mice at any extent. These results demonstrate that ZNF212 might play an important role in maintaining cerebellar Purkinje cell stability. In general, K-ZNF, with a potential transcriptional repression domain, is mainly known as a transcriptional repressor^22^, and the KRAB domain negatively regulates transcription by binding to the corepressor protein KAP1^23^. However, several K-ZNFs have also been introduced as transcriptional activators^24^. For example, ZNF480 is a positive regulator of the MAPK-mediated signaling pathway in vivo^25^. Genome-wide ChIP-sequencing of ZNF263 in K562 cells revealed more than 5,000 binding sites, among which many were transcriptionally activated^26^. ZNF202 has a SCAN domain, which prevents the binding of KAP1 and interacts with the co-activator^27^. ZNF224 interacts with the molecular partner WT1 and acts as a co-activator and has a pro-apoptotic role^28^. ZNF307 activates MDM2 and EP300 gene expression, resulting in p53 degradation^29^. The transactivation role of ZNF398/ZER6 is repressed in the presence of ERα^30^. Notably, ZNF212 upregulates PLD3 at the transcriptional level. K-ZNFs clustered on chromosome 7, including ZNF777, ZNF212, and ZNF398, have a weak binding affinity for KAP1^31^, providing evidence of the molecular mechanism by which ZNF212 may act as a transcriptional activator.

Alcohol-induced cerebellar degeneration is one of the most common forms of cerebellar ataxia^32^. However, it remains unclear which mechanism is involved in alcohol metabolism that contributes to cerebellar degeneration. The proposed pathophysiological mechanisms of alcohol toxicity include excitotoxicity, dietary factors, oxidative stress, compromised energy production, and cell death^33^. Indeed, alcohol-related cerebellar degeneration results in cerebellar atrophy^34^. In ataxic alcoholics, 42% atrophy has been found in the vermal white matter; loss of Purkinje cells and impaired dendritic networks are also commonly found in the molecular layer. Notably, the withdrawal of ethanol after a long period of consumption causes Purkinje cell loss^34^. In alcohol-induced ataxia mice, Zfp212 and Pld3 levels were reduced, indicating that modulation of the ZNF212 and PLD3 pathways can be utilized to develop therapeutic approaches to alcohol-medicated cerebellar abnormalities in humans in future studies.

In this study, we found that the novel K-ZNF protein Zfp212 was highly expressed in the Cb and that the loss of Zfp212 led to Purkinje cell death and ataxia-like behavioral deficits. We identified *PLD3* as a ZNF212 target gene and validated the reduction of PLD3 in the Cb of Zfp212-KO mice. Notably, significant downregulation of Zfp212 and PLD3 was observed in the Cb of alcohol-induced ataxia mice, suggesting that ZNF212 is an essential protein that maintains Purkinje cell health and regulates the level of PLD3 in the Cb.

## Methods

### Animals

All animal experiments were approved by the Sungkyunkwan University Ethical Committee in accordance with international guidelines (SKKUIACUC2020-11–13-1). C57BL/6 background mice were obtained from Orient (Suwon, Korea) and maintained at 12-h dark/light cycles in air-controlled rooms, with access to food and water ad libitum. All efforts were designated to minimize animal suffering and to reduce the number of animals used. This study was carried out in compliance with the ARRIVE guidelines.

To create *Zfp212* knockout (Zfp212-KO) mice, *Zfp212* targeting guide RNA (gRNA) and recombinant Cas9 protein (ToolGen, Korea) was injected into fertilized C57BL/6 mouse eggs. Injected eggs were then implanted into ICR surrogate mothers, and all pups were back-crossed with WT C57BL/6 mice. Heterogeneous mice were crossed to obtain homogeneous Zfp212-KO mice.

### Multiple sequence alignment

Polypeptide sequences of ZNF212/Zfp212 (mouse homolog of human ZNF212) of multiple organisms were obtained from UniProt (https://www.uniprot.org/). Amino acid sequences of ZNF212/Zfp212 from each organism were then inserted into the software Jalview 2.11.0. Multiple sequence alignment was calculated using Jalview software according to the program’s protocol.

### Cell culture and transfection

HEK293, SH-SY5Y, and HT22 cell lines (ATCC, Manassas, VA) were used in this study. Cells were cultured in Dulbecco’s modified Eagle’s medium supplemented with 10% fetal bovine serum (Corning, USA) and 1% penicillin/streptomycin and maintained at 37 °C in a 5% CO_2_ incubator. Plasmid DNA was transfected using TransIT-X2 Transfection Reagent (MIR6003, MirusBio, USA) according to the manufacturer's protocol. Small interfering RNA (siRNA) was transfected with RNAi max (Invitrogen, USA) according to the manufacturer's protocol.

### Immunocytochemistry

Approximately 5 × 10^4^ HEK293 cells were seeded onto polylysine-coated sterile glass coverslips in a 24-well culture plate. After attachment, the cells were washed once with PBS and fixed in 3% paraformaldehyde (PFA; w/v) for 20 min. The fixed cells were washed three times with PBS before permeabilization in 0.2% (v/v) Triton X-100 in PBS for 5 min. Blocking was then carried out with 5% goat serum in PBS for 1 h. Cells were incubated with primary antibodies overnight at 4 °C and secondary antibodies for 1 h at 25 °C. Immunofluorescent images were acquired using a Leica fluorescence microscope (CTR6000, Leica, Germany).

### Histochemistry

Mice were anesthetized with pentobarbital (50 mg/kg, intraperitoneal injection) and perfused with ice-cold PBS and 4% PFA through cardiac puncture. Perfused brains were harvested and incubated in 4% PFA for 48 h at 4 °C, followed by incubation in 30% sucrose/PBS for 24 h for cryoprotection. The brains were sliced into 35 μm-thick sections with a microtome (Thermo Fisher, USA) and permeabilized with 0.25% Triton X-100 in PBS for 30 min.

### Nissl staining

Sections were mounted on glass slides (Superfrost Plus, Thermo Scientific) and washed three times in 95% ethanol for 2 min, followed by incubation in xylene for 5 min. Slides were washed in 100%, 95% ethanol, and water for 5 min and 2 min, respectively, followed by staining in Nissl solution for 5 min. Stained sections were washed three times for 2 min in water and incubated in a formalin acetic solution for 10 min. Three washes in water for 2 min and two washes in 100% ethanol for 5 min were conducted followed by fixing twice in xylene for 10 min. Sections were mounted with DPX mountant (Sigma) and covered with a coverglass (Marienfeld). Stained images were acquired using a Leica microscope (CTR6000, Leica, Germany). Total numbers of Nissl-stained Purkinje cells in cerebellar lobules were counted using Image J software (NIH, USA). For Nissl counting, a cell was defined as a bright blue-stained nucleolus^4^. Counted cells were quantified from at least 3 independent experiments.

### Immunofluorescence

Slices were blocked in 10% normal serum (goat or horse)/PBS. The tissue was probed with primary antibodies overnight at 4 °C and Alexa Fluor fluorescence secondary antibodies (Thermo Fisher Scientific, USA) for a matching host for 1 h at RT. Stained tissues were mounted with Ultracruz mounting medium (Santa Cruz Biotechnologies, USA) containing DAPI. Immunostained images were acquired using a laser scanning confocal microscope equipped with ZEN 2000 Light Edition (LE) software (LSM 710, Carl Zeiss, Germany).

### DAB staining

Brain sections were incubated with primary antibodies overnight at 4 °C and visualized with biotinylated goat anti-rabbit IgG followed by streptavidin-conjugated horseradish peroxidase (Vector Laboratories). Positive immunostaining was visualized with 3,3-diaminobenzidine (Sigma) after reaction with hydrogen peroxide (Vectastine ABC kit, Vector Laboratories). Stained sections were mounted onto slides and analyzed using a Leica microscope (CTR6000, Leica, Germany). Total numbers of calbindin-stained Purkinje cells in cerebellar lobules I/II were counted using Image J software (NIH, USA). Counted cells were quantified from at least 3 independent experiments^4^.

### Golgi staining

Briefly, the mice were anesthetized with pentobarbital (50 mg/kg, intraperitoneal injection) and perfused with PBS followed by fixing with 4% paraformaldehyde for brain harvesting. The brains were immersed in the impregnation solution at RT for 2 weeks then transferred to solution C for 72 h. Brains were then sliced to a thickness of 100 μm using a microtome (Thermo Fisher, USA). Cut sections were mounted onto a glass slide (Superfrost^R^ Plus, Thermo Scientific) and dried naturally at RT. Dried sections were stained with the FD Rapid GolgiStain Kit (FD NeuroTechnologies, USA).

### Open-field test

All test sessions were performed during the afternoon hours of the light cycle (10 AM to 3 PM) in the vivarium where the animals were housed. Calibration of the equipment was performed periodically by the manufacturer. To evaluate the locomotor and anxiety-like behavior of Zfp212-KO mice with less stress^35^, an open-field test was performed in an opaque dark-colored plastic box (length × width × height: 50 × 50 × 50 cM). A camera positioned above the box connected to a computer running EthoVision XT software (Noldus, Netherlands) was used to track the mice. The day before the behavioral test was conducted, the mice were habituated to the behavior room. Mice were placed in an empty open-field box and allowed to freely explore the box for 5 min. After the experiment was completed, the open-field box was cleaned and the next mouse was exposed to the same conditions. The total distance traveled, speed, as well as the number of entries to, time spent in, and percentage distance traveled in the corner and center zones were measured.

Each zones are defined as follows. The field was divided into 25 squares, the center 1 square is the Center zone and 8 squares surrounding the center square is the Middle zone. The inner area zone is composed of the center zone and the middle zone. The 16 remaining squares outside of the inner zone are the outer zone.

### Gait analysis

To compare the gait of Zfp212-KO and WT mice, the animals were allowed to walk along a runway (length × width: 50 × 10 cM) with 10 cM-high walls, reaching an enclosed box. To obtain footprints, the hindfeet and forefeet of the mice were painted with blue and red nontoxic paints, respectively. All mice had three training runs and were then given one run per day on a white sheet of paper. The footprint patterns were analyzed with four parameters; hind-stride and front-stride length were measured as the average distance of forward movement between each stride, and hind-base and front-base widths were measured as the average distance between the left and right footprints. For each step parameter, three values were measured from each run, excluding footprints made at the beginning and end of the run where the animal was initiating and finishing movement, respectively^36^.

### Rotarod test

To assess motor coordination and balance, an accelerating rotarod (Model No. LE8505; Harvard Apparatus, USA) was used. For training, mice were placed on a cylinder for 1 min at 4 rpm and slowly accelerated from 4 to 40 rpm over a 5 min test session^37^.

### Purification of GST-ZNF212 recombinant proteins

The pDEST15-GST-ZNF212 plasmids were transformed into BL21 pLys, which were then grown in the presence of IPTG (0.1 mM) for 4 h at 30 °C. Cells were lysed by sonication in TNE buffer (10 mM Tris–HCl, pH 7.4, 200 mM NaCl, 1 mM EDTA) containing 0.1% Triton X-100 and protease inhibitors, and finally centrifuged at 14,000 rpm for 30 min at 4 °C. After centrifugation, the supernatant was recovered, and GST-ZNF212 was purified using glutathione Sepharose 4 B (GE Healthcare). GST protein was also prepared as a control.

### Cyclic amplification and selection of targets (CAST)

We followed the methods of our previously published protocol^4^. Briefly, oligonucleotides containing 26 random nucleotides (CAST26–CTGTCGGAATTCGCTGACGT-(N)26-CGTCTTATCGGATCCTACGT) were used to generate random double-stranded oligomers for the first round of CAST. Random double-stranded oligomers were subjected to pull-down with GST-ZNF212 bound to glutathione sepharose beads in a mixture of 50 mg of BSA and 50 mg of poly(deoxyinosinic-deoxycytidylic) acid (Sigma) in 500 mL of binding buffer containing 10 mM of Tris (pH 7.5), 200 mM of NaCl, 10% glycerol, 50 mM of ZnCl_2_, 1 mM of MgCl_2_, and 1 mM of DTT. The oligonucleotides were extracted from the beads by applying 100 mL of distilled H_2_O, followed by phenol extraction and ethanol precipitation. An elute was used for the subsequent PCR in the presence of 200 pmol of each primer, CAST-N (CTGTCGGAATTCGCTGACG) and CAST-C, with 25 cycles of 1 min at 94 °C, 1 min at 65 °C, and 1 min at 72 °C. Seven rounds of selection were performed. Following seven selections, oligomers were cloned into the pGEM-T Easy vector according to the manufacturer’s protocol (Promega). Sixteen independent clones were sequenced and aligned using the MACAW software.

### Stereotaxic virus injection and surgery

Three-week-old Zfp212-KO mice were used for stereogenic injection. AAV-GFP and AAV-ZNF212 viruses were purchased from Vector Biolabs (USA). Anesthetized mice with pentobarbital (50 mg/kg, intraperitoneal injection) were injected in two positions in the Cb at the following coordinates: DV: − 1.5 AP: − 5, ML: − 1.5/1.5. After 6 weeks, the mice were subjected to behavioral tests and biochemical experiments.

### Immunoblotting

Cells and homogenized tissue were mixed with RIPA buffer (Thermo Scientific, USA) with 100 × protease inhibitor cocktail (GeneDEPOT, USA), and concentrations were measured by BCA assay. Protein lysate plus 2 × Laemli sample buffer (Bio-Rad, USA) was boiled at 97 °C for 10 min, separated by SDS-PAGE, and transferred to nitrocellulose membranes (Bio-Rad, USA). Membranes were blocked in 5% skim milk/TBST and incubated with 1/3000 diluted primary antibodies and 1/10,000 diluted secondary antibodies. ECL reagent (Thermo Scientific, USA) was used to obtain fluorescence signals from the X-ray films. The antibodies used are listed in Additional file 1, Table S1.

### Reverse transcription-quantitative real-time polymerase chain reaction

We used the Superscript III First-strand synthesis system (18080051, Invitrogen, USA) for the production of cDNA from RNA extracted from cells and tissues. Two-step qPCR was performed using 2 × SYBR green (Qiagen, USA) and Rotor-Gene Q thermal cycler (Qiagen, USA) according to the manufacturer’s protocol. Primer sequences were designed at the Primer3 site (http://bioinfo.ut.ee/primer3/), and their lists are described in Additional file 1, Table S2.

### Traditional cloning

Traditional cloning using restriction enzymes and T4 DNA ligase was used to clone the FLAG-tagged ZNF212 expression vector and luciferase vector containing the *Pld3* promoter. The human ZNF212 open reading frame (ORF) construct was purchased from Origene (USA), and the promoter of human *Pld3* originated from the genomic DNA of the human SH-SY5Y cell line. Human ZNF212 ORF and PLD3 promoters were amplified by PCR (35 cycles) using a KAPA HIFI PCR kit (KAPAbiosystem, USA). Amplified PCR products were cloned into the cut and ligation protocols. Amplified DNA fragments and empty vectors (pCMV-tag2A and pGL3-basic, respectively) were cut using restriction enzymes (NEB, USA) at 37 °C for 3 h and separated on a 0.8% agarose gel (Bioshop, USA)/0.5% tris–acetate EDTA buffer, and then purified with a Gel/PCR clean-up kit (Intron Biotechnology, Korea) according to the manufacturer's protocol. Purified DNA was ligated with T4 DNA ligase (NEB, USA) at 16 °C overnight and transformed into DH5alpha competent cells (Enzynomics, Korea). Cloned plasmids were obtained using the Miniprep kit (Intron Biotechnology, Korea) and sequenced (Cosmogenetech, Korea) for validation.

### Gateway™ cloning

To clone GFP-ZNF212 and GST-ZNF212, the Gateway™ cloning method (Invitrogen, USA) was used. Amplified human ZNF212 ORF was cloned into the pCR8/GW topo vector (Invitrogen, USA) to obtain entry vectors. Entry vectors were verified by DNA sequencing (Cosmo Genetech, Korea). Entry and DEST vectors (pcDNA6.2/N-emGFP and pDEST15-GST, respectively) were recombined with LR clonase II (Invitrogen, USA) according to the manufacturer's protocol.

### Site-directed mutagenesis

Plasmids were amplified by PCR (14 cycles) with mutant primers and cut with DpnI enzyme (NEB, USA) followed by enzyme deactivation at 85 °C. After enzyme deactivation, the plasmids were transformed into DH5alpha competent cells (Enzynomics, Korea). Cloned plasmids were obtained using a Miniprep kit (Intron Biotechnology, Korea). Mutants were confirmed by DNA sequencing (Cosmo Genetech, Korea). Primer lists used in cloning are provided in Table S3.

### Chromatin Immunoprecipitation assay

SH-SY5Y cells were fixed with 1% formaldehyde for 10 min at RT, and the powdered mouse Cb was suspended in 1% formaldehyde in PBS for 20 min at RT. Chromatin immunoprecipitation was performed using the SimpleChIP Enzymatic Chromatin IP kit (Cell Signaling Technology, USA) according to the manufacturer’s protocol with modifications^4^. Pre-cleared chromatin was incubated with antibodies against FLAG, ZNF212, or rabbit IgG (rIgG)-conjugated agarose beads, followed by three washes. Elutes were subjected to reverse cross-linking, and DNA was recovered using a spin column.

### Luciferase assay

A set of plasmids was transfected into SH-SY5Y cells and harvested with passive lysis buffer at 36 h post-transfection. Luciferase assay kit (Promega, USA) was used for the promoter assay, and luciferase signals were obtained with Glomax (Promega, USA) according to the manufacturer's protocol.

### Statistical analysis

Data are presented as the mean ± standard error of the mean (SEM) for at least three independent experiments. Student’s t-test was used to compare the statistical significance between the two groups. One-way ANOVA with Tukey’s post-hoc test was used to compare the values of multiple groups. Kaplan–Meier method was used to visualize the survival curve of the mice. Statistical analysis was conducted with GraphPad Prism 8.

## Supplementary Information

Supplementary Information.

## Data Availability

The datasets used and/or analyzed during the current study are available from the corresponding author upon reasonable request.
