# Supplementary material for: Loss of zinc-finger protein 212 leads to Purkinje cell death and locomotive abnormalities with phospholipase D3 downregulation
Source: Sci Rep. 2021 Nov 23;11:22745. doi: 10.1038/s41598-021-02218-x (PMC8610974; doi:10.1038/s41598-021-02218-x)
Supplement: Supplementary file 1 — Supplementary Information. [file 41598_2021_2218_MOESM1_ESM.docx]

**Supplementary Information**

**Loss of zinc-finger protein 212 leads to Purkinje cell death and locomotive abnormalities with phospholipase D3 downregulation**

Rin Khang^1,2^, Areum Jo^1,2^, Hojin Kang^1,2^, Hanna Kim^1,2^, Eunsang Kwag^1,2^, Ji-Yeong Lee^1,2^, Okjae Koo^3,*^, Jinsu Park^4^, Hark Kyun Kim^4^, Dong-Gyu Jo^4,5^, Inwoo Hwang^2,6^, Jee-Yin Ahn^2,6,7^, Yunjong Lee^1,7^, Jeong-Yun Choi^1,7^, Yun-Song Lee^1,7^, and Joo-Ho Shin^1,2,7,#^

**Supplementary Tables**

Table S1. Antibodies used

Table S2. Sequences of RT-qPCR primers

Table S3. Information on primers used for plasmid construction, genotyping, and ChIP

**Supplementary Figures**

Figure S1. ZNF212 is highly conserved in mammals

Figure S2. ZNF212 localizes in the nucleus and expresses at the postnatal stage

Figure S3. Generation of Zfp212 knockout (KO) mice

Figure S4. The loss of Purkinje neurons in the cerebellum of 18-month-old Zfp212-KO mice

Figure S5. ZNF212 regulates PLD3 expression in HT22 cells

Figure S6. Zfp212 occupies the promoter of Pld3 in the mouse cerebellum

**Table S1. Antibodies used**

| No. | Name | Host | Company |
| --- | --- | --- | --- |
| 1 | FLAG | Mouse Monoclonal | Sigma Aldrich |
| 2 | β-actin (HRP-conjugated) | Mouse Monoclonal | Abcam |
| 3 | ZNF212 | Rabbit polyclonal | Atlas Antibodies |
| 4 | Calbindin | Rabbit monoclonal | Abcam |
| 5 | Calbindin | Mouse monoclonal | Sigma Aldrich |
| 6 | GAD65/67 | Rabbit polyclonal | Novus Biology |
| 7 | NeuN | Mouse monoclonal | Abcam |
| 8 | GFAP | Mouse monoclonal | Abcam |
| 9 | PLD3 | Rabbit polyclonal | Atlas Antibodies |
| 10 | GFP | Mouse monoclonal | Enzo Life Sciences |
| 11 | Rabbit IgG (HRP-conjugated) | Goat polyclonal | Bethyl Laboratories |
| 12 | Mouse IgG (HRP-conjugated) | Sheep | GE Healthcare |
| 13 | Rabbit IgG (Biotin-SP conjugated) | Goat polyclonal | Jackson ImmunoResearch |
| 14 | Mouse IgG (AlexaFluor 488) | Goat | ThermoFisher Scientific |
| 15 | Rabbit IgG (AlexaFluor 488) | Goat | ThermoFisher Scientific |
| 16 | Mouse IgG (AlexaFluor 594) | Goat | ThermoFisher Scientific |
| 17 | Rabbit IgG (AlexaFluor 594) | Goat | ThermoFisher Scientific |

**Table S2. Sequences of RT-qPCR primers**

| No. | Name | Sequence | Product size (bp) |
| --- | --- | --- | --- |
| 1 | mRpl32-RTq-f | gcccaagatcgtcaaaaaga | 217 |
| 2 | mRpl32-RTq-r | attgtggaccaggaacttgc |  |
| 3 | mActb-RTq-f | cctctatgccaacacagtgc | 206 |
| 4 | mActb-RTq-r | cctgcttgctgatccacatc |  |
| 5 | mAdap1_RTq-f | aggggaagtcttcattggca | 220 |
| 6 | mAdap1_RTq-r | ttccactgcgtactcctgag |  |
| 7 | mAtp1a3_RTq-f | cgacatcatgaagaggcagc | 197 |
| 8 | mAtp1a3_RTq-r | cagtgcgatcatcccagttg |  |
| 9 | mAtxn2_RTq-f | cagtcttcaccatccaccct | 247 |
| 10 | mAtxn2_RTq-r | tgtactgaagggtgcgtcat |  |
| 11 | mAtxn7_RTq-f | caacagcaccatcttcccac | 170 |
| 12 | mAtxn7_RTq-r | gagagagagagtggagtcgc |  |
| 13 | mAtxn7l3_RTq-f | gccgttcttccagaggtaga | 183 |
| 14 | mAtxn7l3_RTq-r | ccgtgattcagagctgttgg |  |
| 15 | mBean1_RTq-f | atgaggaatgcatgggacca | 206 |
| 16 | mBean1_RTq-r | ataaggtggcagtgtgtcca |  |
| 17 | mCacna1a_RTq-f | ctctgggccgatacactgat | 158 |
| 18 | mCacna1a_RTq-r | atgatgatggtggtggtggt |  |
| 19 | mCacna1g_RTq-f | ttcaaacttgtggccttcgg | 154 |
| 20 | mCacna1g_RTq-r | tgatacggatgatggtgggg |  |
| 21 | mDab1_RTq-f | gggattgatgaagtgtccgc | 241 |
| 22 | mDab1_RTq-r | atccgaaagcccgatgatct |  |
| 23 | mItpr1_RTq-f | gaaagccaaagagcccacaa | 188 |
| 24 | mItpr1_RTq-r | cagcgctcatctctaggtca |  |
| 25 | mKcnc3_RTq-f | tgatcctgacgacatcctgg | 200 |
| 26 | mKcnc3_RTq-r | tgttgacaatgacaggcacg |  |
| 27 | mKcnd3_RTq-f | gtctacggatcctgggctac | 222 |
| 28 | mKcnd3_RTq-r | ctgcgattgtcttaggcacc |  |
| 29 | mPrkcg_RTq-f | actccacctttcagactccg | 171 |
| 30 | mPrkcg_RTq-r | agatgatgccctggttgtga |  |
| 31 | mSptbn2_RTq-f | ggtgcttgtgtttgggagag | 250 |
| 32 | mSptbn2_RTq-r | ctgtttcctccgttcctcct |  |
| 33 | mTmem240-RTq-f | tggacgcctctgagaactac | 177 |
| 34 | mTmem240-RTq-r | aggtccacgagccatcatag |  |
| 35 | mTtbk2_RTq-f | ccaccatgaccagaggagtt | 167 |
| 36 | mTtbk2_RTq-r | tagagctggttgagtggtgg |  |
| 37 | mGrid2_RTq-f | ggatgcagctgtgttggaat | 176 |
| 38 | mGrid2_RTq-r | gaatgtccatgtcgccactc |  |
| 39 | mElovl4_RTq-f | ttccttcacgtgtaccacca | 197 |
| 40 | mElovl4_RTq-r | agcatggtcaggtatcgctt |  |
| 41 | mPld3_RTq-f | atgaactacctgcccaccat | 183 |
| 42 | mPld3_RTq-r | gttgtcatgaagtgcagcca |  |
| 43 | mAfg3l2-RTq-f | aggaaggttacccagagtgc | 224 |
| 44 | mAfg3l2-RTq-r | ttctccacgtcagccttctt |  |
| 45 | mAtxn1-Rtq-f | gctgccgccatatttcatga | 158 |
| 46 | mAtxn1-Rtq-r | ggctctcctcgattctctcc |  |
| 47 | mAtxn3-Rtq-f | gtccaacagatgcatcgacc | 226 |
| 48 | mAtxn3-Rtq-r | gcatactgagctgaatggcc |  |
| 49 | mAtxn10-RTq-f | tcagagtggccgttcttgat | 153 |
| 50 | mAtxn10-RTq-r | atcctttgtcagctgctcct |  |
| 51 | mCcdc88c-RTq-f | cctctcaggctctcagatcg | 236 |
| 52 | mCcdc88c-RTq-r | ggtggctgaggttgaaaagg |  |
| 53 | mdnmt1-RTq-f | tgtgaacgagaccctgtacc | 193 |
| 54 | mdnmt1-RTq-r | gtgggtattctcaggcctgt |  |
| 55 | mEef2-RTq-f | tctgacaaaggccgcttcta | 158 |
| 56 | mEef2-RTq-r | tagcggcccatcatcagaat |  |
| 57 | mElovl5-RTq-f | tacatcactcaagggcagct | 180 |
| 58 | mElovl5-RTq-r | agaggcccctttcttgttgt |  |
| 59 | mFat2-RTq-f | cagaatcacggcgtcagatg | 215 |
| 60 | mFat2-RTq-r | ccggtccatgcagagagtat |  |
| 61 | mFgf14-RTq-f | agggagtgaagacagggttg | 235 |
| 62 | mFgf14-RTq-r | tgagctgctggtttggtttt |  |
| 63 | mGrm1-RTq-f | acctctgatgtagtgcgcat | 221 |
| 64 | mGrm1-RTq-r | ttacaggccgtctcattggt |  |
| 65 | mIfrd1-RTq-f | gacaagagaaagcagcggtc | 172 |
| 66 | mIfrd1-RTq-r | ggtactgcatccctgatcca |  |
| 67 | mMme-RTq-f | ttcctcaggccgaaatcaga | 181 |
| 68 | mMme-RTq-r | ccagtcaacgaggtctccat |  |
| 69 | mNop56-RTq-f | gagactggagagatcccacg | 170 |
| 70 | mNop56-RTq-r | ctgttttctgacgaggccag |  |
| 71 | mPdyn-RTq-f | tgaatgatgaagccgcacag | 240 |
| 72 | mPdyn-RTq-r | catagcgcttctggttgtcc |  |
| 73 | mPPP2r2b-RTq-f | gatcctgccaccatcacaac | 234 |
| 74 | mPPP2r2b-RTq-r | catgttggctggcttgatgt |  |
| 75 | mTgm6-RTq-f | gcagggcaaatatggaggtg | 193 |
| 76 | mTgm6-RTq-r | gtgggctgagttgaagttgg |  |
| 77 | mTbp-RTq-f | gcagcctcagtacagcaatc | 198 |
| 78 | mTbp-RTq-r | ctgcggtacaattccagagc |  |
| 79 | mTrpc3-RTq-f | cgcctcggacagatttgaag | 205 |
| 80 | mTrpc3-RTq-r | cacattccacaactgcacga |  |
| 81 | mTubb4a-RTq-f | ccccgtctccacttcttcat | 238 |
| 82 | mTubb4a-RTq-r | aactgctgttcttgctctgc |  |
| 83 | mZfp212RTq-f | acttctgggtccttcgtctg | 170 |
| 84 | mZfp212RTq-r | caccagcgtctcatagttgc |  |

**Table S3. Information on primers used for plasmid construction, genotyping, and ChIP**

| No. | Name | Sequence | Usage |
| --- | --- | --- | --- |
| 1 | NotI-znf212-f | GCG GCC GCT atg gcg gag tcg gcg | pCMV-tag2A ZNF212 |
| 2 | XhoI-znf212-r | CTC GAG tta aag cag gcc att ggg |  |
| 3 | pENTR ZNF212-f | ATG GCG GAG TCG GCG | pENTR-ZNF212 |
| 4 | pENTR ZNF212-r | aag cag gcc att ggg |  |
| 5 | HindIII-hPLD3p-f | AAG CTT gaa ttg ctc gaa cca ggg agg | pGL3-hPLD3p |
| 6 | HindIII-hPLD3p-r | AAG CTT gct tca tct tcc ctc aag gc |  |
| 7 | hPLD3IRLS1del-f | ggggtctccagtgtccagccggggc | pGL3-hPLD3p IRLS1 deletion |
| 8 | hPLD3IRLS1del-r | gccccggctggacactggagacccc |  |
| 9 | hPLD3IRLS2del-f | caaataccaaaatcgactccaacagaaaattcacc | pGL3-hPLD3p IRLS2 deletion |
| 10 | hPLD3IRLS2del-r | ggtgaattttctgttggagtcgattttggtatttg |  |
| 11 | hPLD3IRLS3del-f | gttttattaatttcatttatgtttttgactaggtaatgcatgtcc | pGL3-hPLD3p IRLS3 deletion |
| 12 | hPLD3IRLS3del-r | ggacatgcattacctagtcaaaaacataaatgaaattaataaaac |  |
| 13 | hPLD3IRLS4del-f | ggttccttttgtcctgtggcctccc | pGL3-hPLD3p IRLS4 deletion |
| 14 | hPLD3IRLS4del-r | gggaggccacaggacaaaaggaacc |  |
| 15 | zfp212 KO seq-f | tacagCACAGGAGGAAACGG | Genotyping |
| 16 | Zfp212 KO seq-r | CAAGGGACCCAGATACctgc |  |
| 17 | hPLD3p-ChIP-f | gga ggg atg aga gag gaa ag | ChIP |
| 18 | hPLD3p-ChIP-r | ggt cag agg aga tgg tga at |  |
| 19 | hPLD3p-Intron-f | cagacacgcagaggatcatg | ChIP |
| 20 | hPLD3p-Intron-r | ctgggctcaagcaatcctcc |  |
| 21 | mPld3p-ChIP-f | ccagcacgctctatttccag | ChIP |
| 22 | mPld3p-ChIP-r | gagactgaggcaggaggatat |  |
| 23 | mPld3p-Intron-f | cgctggtcactcttccatctttg | ChIP |
| 24 | mPld3p-Intron-r | ggagatggaaacaggaagtacaag |  |

**Supplementary Figure legends**

**
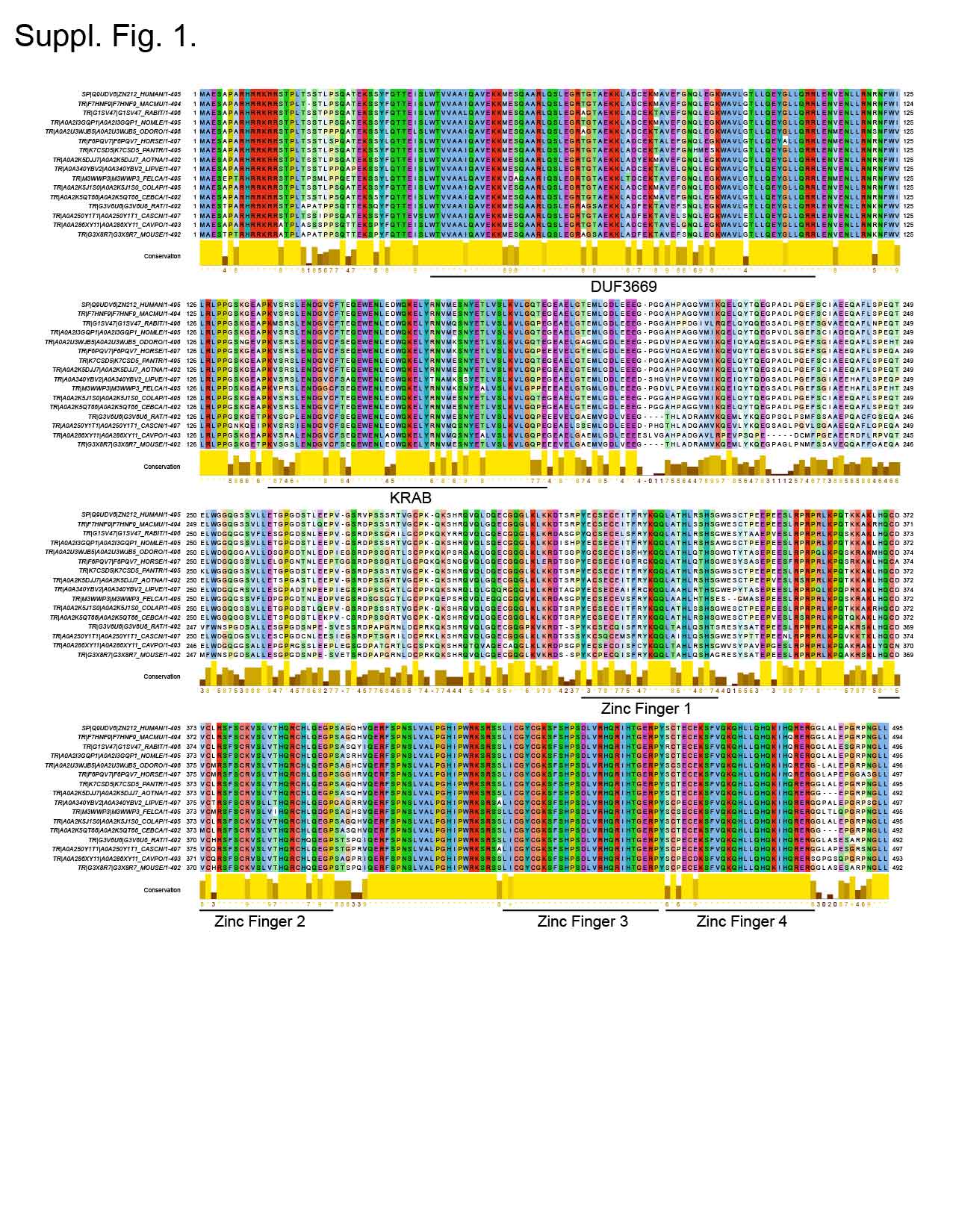
**

**Figure S1. ZNF212 is highly conserved in mammals**

Multiple sequence alignment of mammalian ZNF212. Polypeptide sequences of ZNF212/Zfp212 were obtained from UniProt (https://www.uniprot.org/). Amino acid sequences of ZNF212/Zfp212 were inserted into Jalview 2.11.0 (https://www.jalview.org/) and aligned.


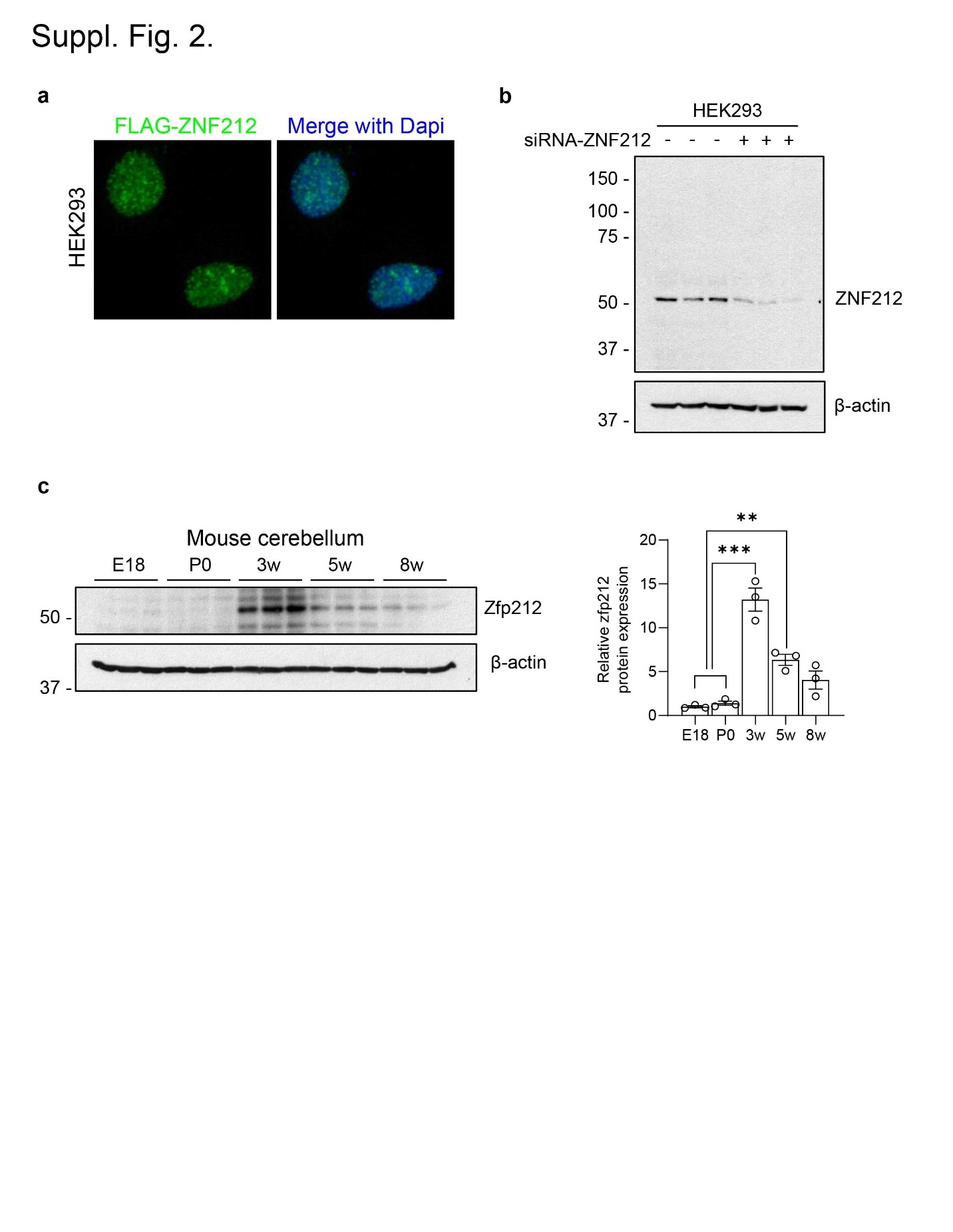


**Figure S2. ZNF212 localizes in the nucleus and expresses at the postnatal stage**

**a** Immunofluorescence analysis with HEK293 cells transfected with Flag-tagged ZNF212.

**b** Validation of ZNF212 antibody with HEK293 cells transfected with siRNA-ZNF212.

**c** Immunoblot of Zfp212 in the mouse cerebellum at different developmental stages (E18: embryonic day 18, P0: postnatal day 0, 3w: 3-week-old, 5w: 5-week-old, 8w: 8-week-old). Zfp212 expression was quantified (right panel, *n* = 3).


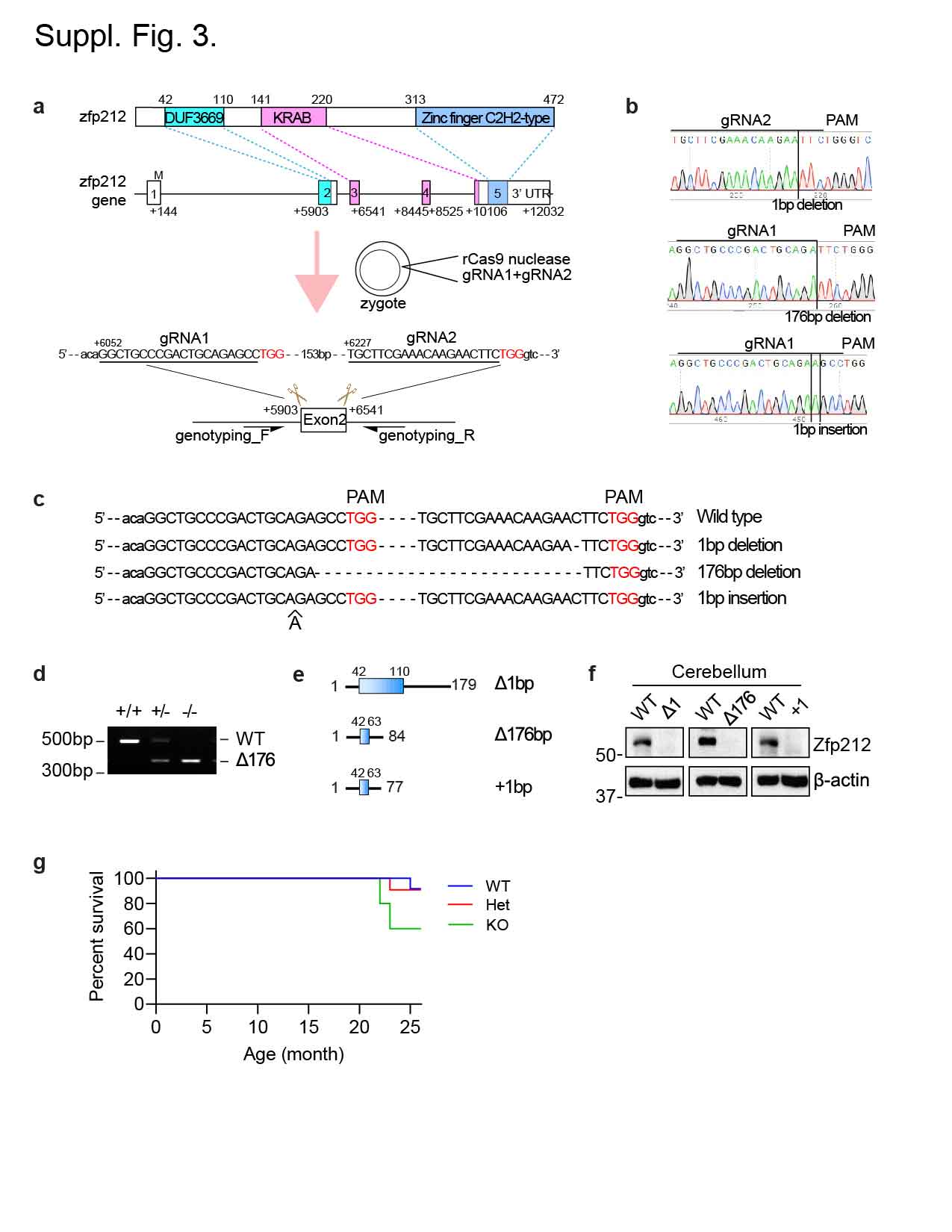


**Figure S3. Generation of Zfp212 knockout (KO) mice**

**a** Schematic illustration for generating Zfp212 KO mice with CRISPR-Cas9. Recombinant Cas9 protein and two guide RNAs were microinjected into fertilized mouse zygotes.

**b** Three Zfp212-KO mouse lines were validated by DNA sequencing.

**c** Comparison of the DNA sequence from three different Zfp212-KO mouse lines

**d** PCR genotyping of the Zfp212-KO (Δ176) line.

**e** Prediction of Zfp212 products from three different Zfp212-KO mouse lines.

**f** Immunoblot analysis of Zfp212 in the cerebellum of three different Zfp212-KO mouse lines.

**g** Survival curves of three different Zfp212-KO mouse lines. Survival curves were calculated by Kaplan-Meier survival analysis (WT, *n* = 12; Het, *n* = 11; KO, *n* = 7).


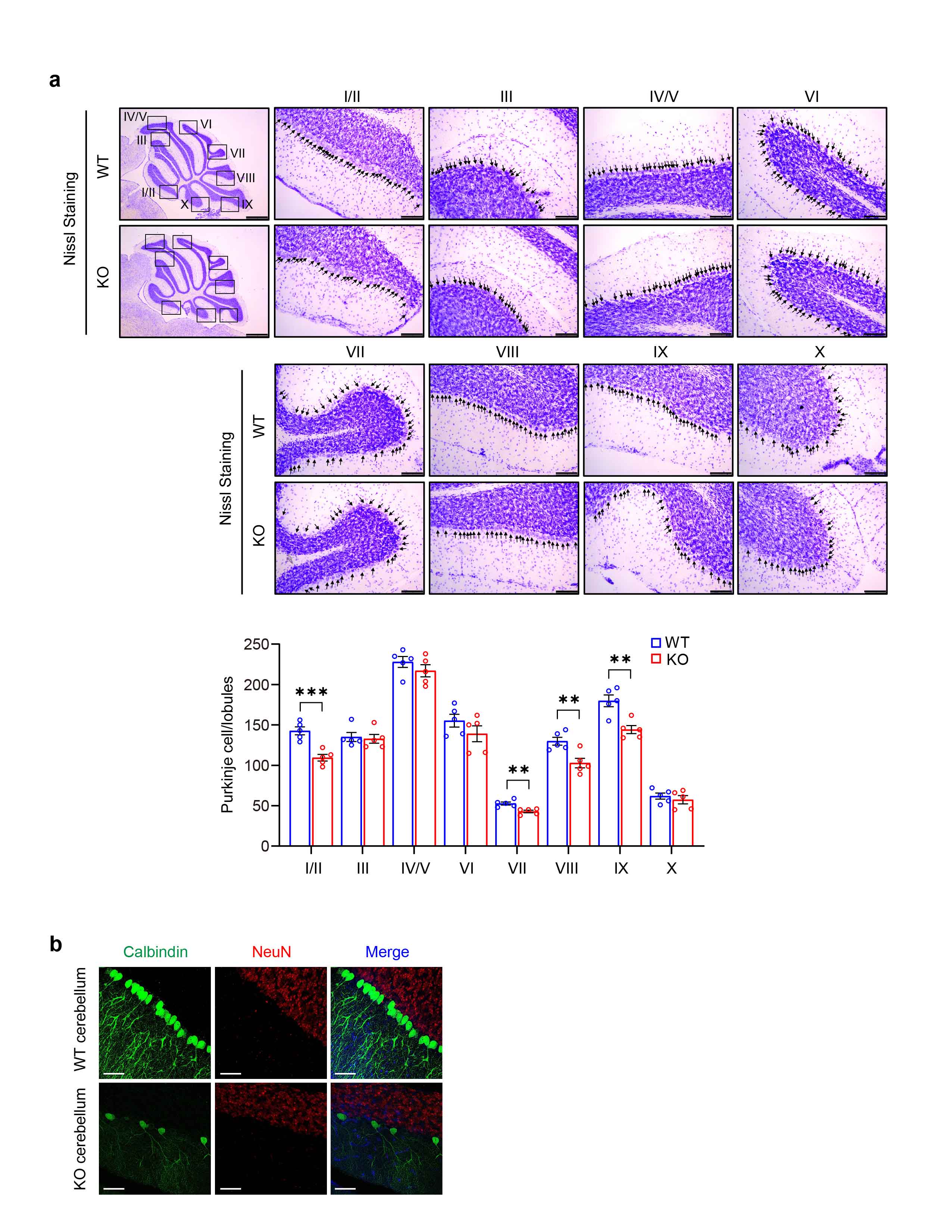


**Figure S4. The loss of Purkinje neurons in the cerebellum of 3- and 18-month-old Zfp212-KO mice**

**a** Representative image of Nissl staining in the cerebellum of Zfp212-WT and -KO mice at 3 months of age. Black arrows indicate Purkinje cells in the lobules. Scale bars = 800 μm at the original image and 100 μm at the enlarged image, respectively.

**b** Representative image of the immunofluorescence staining of neuronal markers in the cerebellum of 18-month-old Zfp212-WT and -KO mice. Images were processed with ZEN 2009 Light Edition software (https://www.zeiss.com/microscopy/int/products/microscope-software/zen-lite.html). Scale bars = 50 μm.


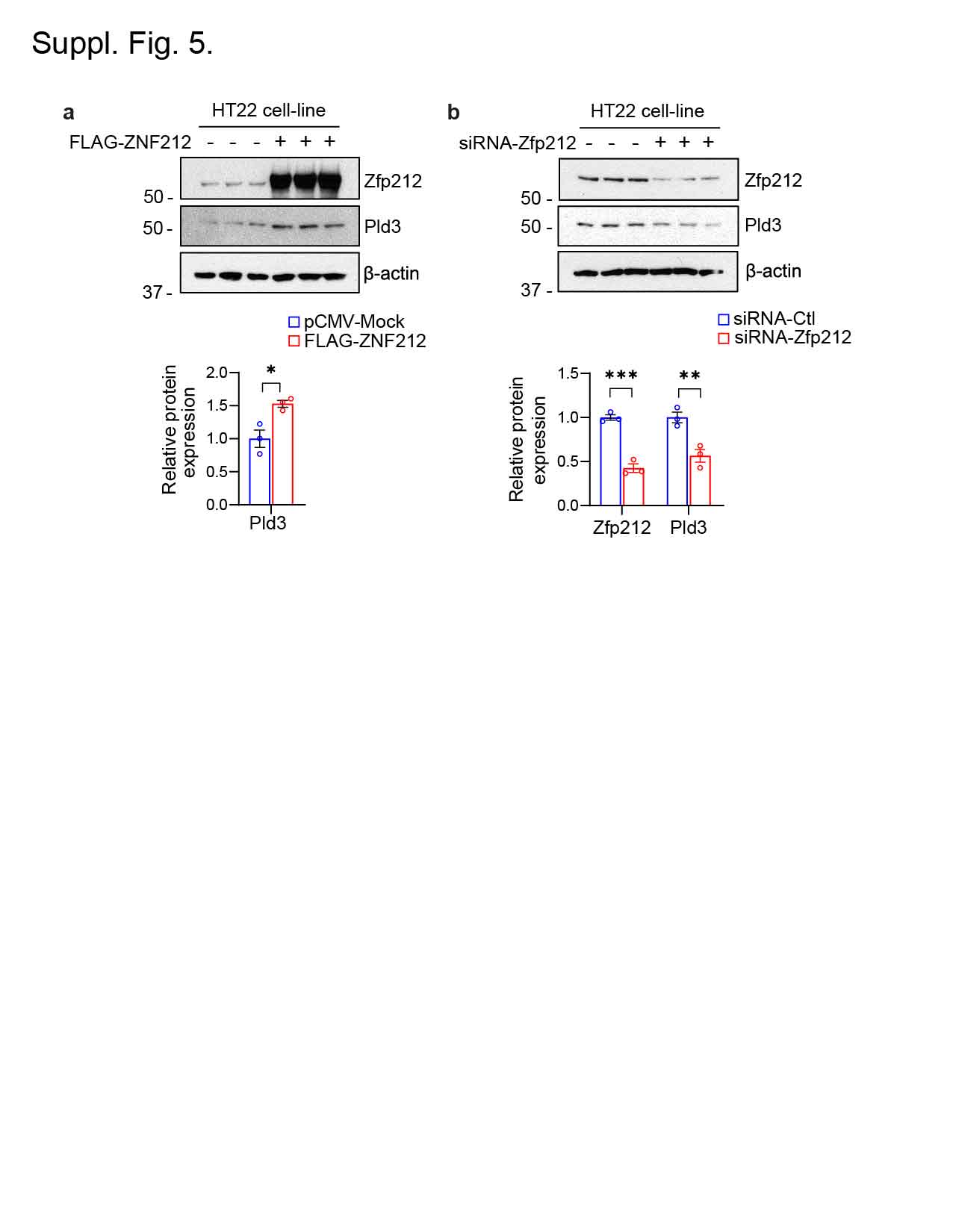


**Figure S5. ZNF212 regulates PLD3 expression in HT22 cells**

**a** Immunoblot analysis of ZNF212 and PLD3 in HT22 cells overexpressing Flag-tagged ZNF212. Quantification is shown at the bottom of the figure (n = 3).

**b** Protein levels of ZNF212 and PLD3 in HT22 cells transfected with siRNA-ZNF212. Quantification is shown at the bottom of the figure (n = 3). Data are expressed as the mean ± SEM. **p* < 0.05; ***p* < 0.01, ****p* < 0.001.


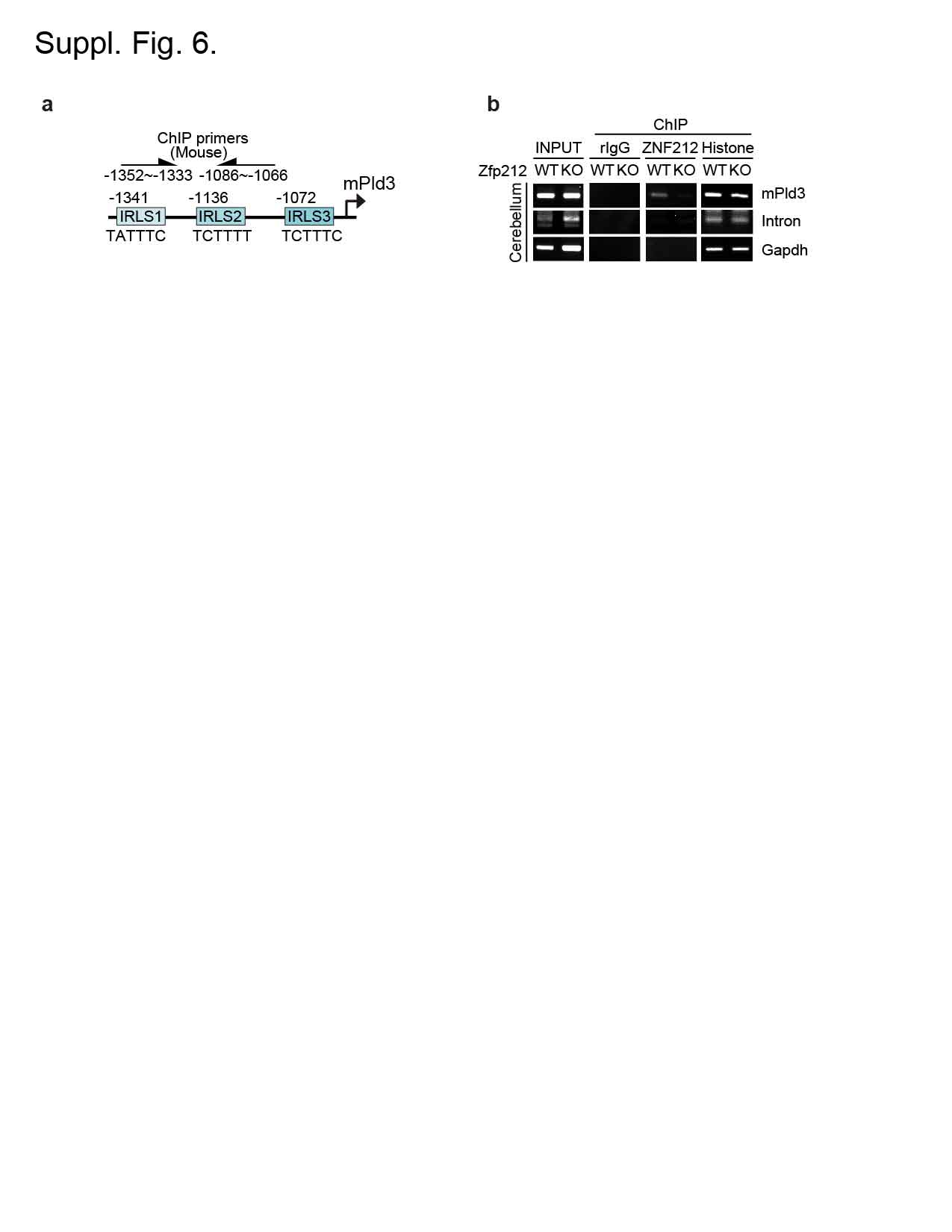


**Figure S6. Zfp212 occupies the promoter of Pld3 in the mouse cerebellum**

**a** Schematic image of the mouse Pld3 promoter containing IRSLs.

**b** ChIP assays with the mouse cerebellum Rabbit IgG (rIgG) and histone antibodies were used as negative and positive controls, respectively.
